# Music reduces pain and increases resting state fMRI BOLD signal amplitude in the left angular gyrus in fibromyalgia patients

**DOI:** 10.3389/fpsyg.2015.01051

**Published:** 2015-07-22

**Authors:** Eduardo A. Garza-Villarreal, Zhiguo Jiang, Peter Vuust, Sarael Alcauter, Lene Vase, Erick H. Pasaye, Roberto Cavazos-Rodriguez, Elvira Brattico, Troels S. Jensen, Fernando A. Barrios

**Affiliations:** ^1^Subdireccion de Investigaciones Clinicas, Instituto Nacional de Psiquiatria “Dr. Ramón de la Fuente Muñiz,”Mexico City, Mexico; ^2^Cátedras, National Council of Science and Technology (CONACYT)Mexico City, Mexico; ^3^Department of Neurology, Faculty of Medicine and University Hospital “Dr. Jose E. Gonzalez” and Neuroscience Unit, Center for Research and Development in the Health Sciences, Universidad Autónoma de Nuevo LeónMonterrey, Mexico; ^4^Music in the Brain, Center of Functionally Integrative Neuroscience, Aarhus UniversityAarhus, Denmark; ^5^Human Performance and Engineering, Kessler FoundationWest Orange, NJ, USA; ^6^Department of Biomedical Engineering, New Jersey Institute of TechnologyNewark, NJ, USA; ^7^Royal Academy of MusicAarhus, Denmark; ^8^Department of Behavioral and Cognitive Neurobiology, Institute of Neurobiology, Universidad Nacional Autonoma de MexicoQueretaro, Mexico; ^9^Department of Psychology and Behavioral Sciences, University of AarhusAarhus, Denmark; ^10^Danish Pain Research Center, Aarhus University HospitalAarhus, Denmark; ^11^Helsinki Collegium for Advanced Studies, University of HelsinkiHelsinki, Finland

**Keywords:** fibromyalgia, music, pain, analgesia, resting state fMRI, BOLD signal, angular gyrus, fALFF

## Abstract

Music reduces pain in fibromyalgia (FM), a chronic pain disease, but the functional neural correlates of music-induced analgesia (MIA) are still largely unknown. We recruited FM patients (*n* = 22) who listened to their preferred relaxing music and an auditory control (pink noise) for 5 min without external noise from fMRI image acquisition. Resting state fMRI was then acquired before and after the music and control conditions. A significant increase in the amplitude of low frequency fluctuations of the BOLD signal was evident in the left angular gyrus (lAnG) after listening to music, which in turn, correlated to the analgesia reports. The *post-hoc* seed-based functional connectivity analysis of the lAnG showed found higher connectivity after listening to music with right dorsolateral prefrontal cortex (rdlPFC), the left caudate (lCau), and decreased connectivity with right anterior cingulate cortex (rACC), right supplementary motor area (rSMA), precuneus and right precentral gyrus (rPreG). Pain intensity (PI) analgesia was correlated (*r* = 0.61) to the connectivity of the lAnG with the rPreG. Our results show that MIA in FM is related to top-down regulation of the pain modulatory network by the default mode network (DMN).

## Introduction

Music-induced analgesia (MIA) is defined as the subjective reduction of pain perception after listening to music (Roy et al., 2008). It has been demonstrated in acute experimental pain (Guétin et al., 2012; Gutgsell et al., 2013; Matsota et al., 2013; Onieva-Zafra et al., 2013; Yeo et al., 2013) and chronic pain diseases such as neuropathic pain, osteoarthritis, and fibromyalgia (FM) (McCaffrey and Freeman, 2003; Özer et al., 2013; Finlay, 2014). MIA seems arise from cognitive and emotional mechanisms elicited by listening to music, such as distraction (Garza-Villarreal et al., 2012), reappraisal (Wiech et al., 2008b), familiarity (Pereira et al., 2011; van den Bosch et al., 2013), emotion (Roy et al., 2012), belief, and reward (Scott et al., 2007; Tracey, 2010; Salimpoor et al., 2013; Hsieh et al., 2014). These factors are likely to contribute to the modulation of pain perception via cortical and subcortical brain areas involved in the pain modulatory system (Bingel and Tracey, 2008). In order to induce a measurable MIA, it is recommended to use music that is pleasant, liked, relaxing and familiar to the person (Mitchell and MacDonald, 2006; Mitchell et al., 2006, 2007).

Recent behavioral studies have shown MIA and other positive effects of music on mood and functional mobility even in individuals who chronically suffer from pain, namely in FM patients (Guétin et al., 2012; Onieva-Zafra et al., 2013; Garza-Villarreal et al., 2014). FM is a chronic pain disease of unknown etiology with a prevalence of 2.1% (female:male ratio of 9:1) according to a recent German survey (Wolfe et al., 2013). FM is characterized by diffuse generalized musculoskeletal pain and increased sensitivity to visual, tactile and auditory stimuli, with depression and fatigue as the most common comorbidities (Geisser et al., 2007). It has been suggested that FM is related to altered somatosensory and nociceptive input to the brain, either via increased sensory input and/or reduced inhibition of the input (Clauw, 2009; Brederson et al., 2011; Smith et al., 2011). This hypothesis has been supported by recent resting state functional magnetic resonance imaging (rsfMRI) studies that found alterations in brain function and connectivity in FM patients (Nebel and Gracely, 2009; Jorge and Amaro, 2012). rsfMRI is an imaging method to evaluate brain function when a subject is not engaged in an explicit task and it has been widely used to study the neurophysiological basis for FM pain and analgesia as it is capable of probing the low-frequency fluctuations (LFFs) of the blood oxygen level-dependent (BOLD) signal across the whole brain and patterns of functional connectivity can be inferred between brain areas across time, without the need of an specific task (Greicius et al., 2003). Some of the advantages of rsfMRI over conventional task-based fMRI in FM pain studies are: (1) usually task-based fMRI studies require somatosensory stimulation to induce pain, whereas rsfMRI is task-free and more tolerable to patients, (2) it is easier to standardize the protocol as it requires no task, making it easier to compare results across studies and populations, (3) the spontaneous pain reported by FM patients is very difficult to assess and to analyze in a task-based form, whereas rsfMRI can capture the dynamic changes in brain activity related to the spontaneous pain.

Several brain areas are involved in the ascending and/or descending pain modulatory systems that are integrated as a feed-back loop, thus both systems are part of the pain experience and modulation. The ascending system conveys the input from the spinal cord to the brainstem [rostroventromedial medulla, and periaqueductal gray (PAG)], cerebellum, subcortical (thalamus, hippocampus, amygdala, hypothalamus, nucleus accumbens), and cortical structures (insula, primary somatosensory cortex, orbitofrontal cortex (OFC), dorsolateral prefrontal cortex, ventromedial prefrontal cortex, ventrolateral prefrontal cortex, medial anterior cingulate cortex, and rostral anterior cingulate cortex). Being part of the descending system, the aforementioned brain areas influence pain perception via top-down mechanisms, involving the brainstem especially the PAG and the rostroventromedial medulla (Tracey and Dickenson, 2012). Using rsfMRI it has been possible to detect functional brain pathology related to the FM chronic pain and analgesia (Napadow et al., 2010, 2012). An experimental pain study found decreased functional connectivity in the descending pain system of FM patients compared to healthy controls (Jensen et al., 2012). Another study found enhanced rsfMRI connectivity in FM patients compared to healthy participants between several brain areas: anterior cingulate cortex (ACC) with the insula and the basal ganglia, primary motor cortex (M1) with supplementary motor area (SMA) and middle prefrontal cortex (mPFC) with posterior cingulate cortex (PCC), as well as reduced connectivity between ACC with amygdala and the PAG, and thalamus with insula and PAG (Cifre et al., 2012). Pujol et al. (2014) also showed reduced connectivity in FM compared to healthy controls between several areas, such as: between PAG and anterior insula, the parietal operculum and S1, and increased connectivity between the parietal operculum and PCC, ACC, and left angular gyrus (lAnG). The PCC and lAnG are part of the default mode network (DMN) (Greicius et al., 2003, 2008), a resting state network described as a “default” state of brain function related to episodic memory, free thinking and preparation for future tasks. Using a power spectrum method for analyzing BOLD signal, a study showed increased power of low frequency fluctuations in S1, SMA, dlPFC, and amygdala in FM patients during resting state (Kim et al., 2013a). In summary, the literature suggests that the ascending and descending pain modulatory systems (i.e., PAG, thalamus, insula, ACC, PCC, dlPFC) may be functionally affected in FM and that it is detectable using rsfMRI.

In a previous behavioral study using a non-pharmacological analgesic intervention, we found that listening to music reduced pain in patients with FM and that the analgesic effect was correlated with increased functional mobility (Garza-Villarreal et al., 2014). The effects of analgesia in FM using fMRI have mostly been studied with task-based (touch or pain) experimental stimuli. For example in a task-based fMRI study showed that pregabalin-induced (pharmacological) analgesia increased the related BOLD signal of a somatosensory stimulus in thalamus, postcentral gyrus, middle frontal gyrus, middle cingulate cortex, inferior parietal lobule, precuneus and insula (Kim et al., 2013b). However, one study showed the effects of a non-pharmacological analgesic intervention in FM using rsfMRI, and they found a reduction of insula connectivity with the DMN post-intervention (Napadow et al., 2012). Therefore, rsfMRI seems to be a promising in measuring and analyzing the effects of MIA in patients with FM.

The amplitude of LFFs [Fractional amplitude of low frequency fluctuations (fALFF)] is an analysis method that aims at measuring resting state BOLD signal changes in terms of power in a specific frequency band within each brain voxel (Zou et al., 2008). By evaluating the amplitude of LFFs of the BOLD signal (0.01–0.08 Hz), this approach has been successful in detecting regional abnormalities in spontaneous BOLD-signal oscillations by measuring the time-series amplitude in each voxel (Biswal et al., 2010; Hong et al., 2013). We decided to apply this method in our study because a connectivity-based approach usually requires the definition of functional networks by various methods, and the chronic pain these patients suffer may have an impact in the shape and temporality of these networks. Instead, a power BOLD-based approach as the fALFF compares the signal amplitude in each voxel across time and does not require network definition. Also, a previous study showed rsfMRI BOLD changes in the power of LFF in FM patients (Kim et al., 2013a).

In this study, we aimed to reveal the neural correlates of MIA in FM by investigating the changes in resting state BOLD signal amplitude and connectivity related to MIA in patients with FM. To this purpose, we performed rsfMRI in 23 FM patients, before and after exposure to music and pink noise. We hypothesized that the analgesic effect would be associated to changes in BOLD signal amplitude and connectivity within the pain modulation systems.

## Materials and methods

### Patients

Twenty-three patients (22 female) with median age of 50 (range: 22–70 years old) were recruited from the Hospital General of the Secretaria de Salud and from a FM help group, both in the city of Queretaro, Mexico. The higher incidence of FM in females is the reason we could not balance the sample's sex. The FM patients attended to a two-part experiment: behavioral and fMRI. The results of the behavioral part of the experiment were reported in Garza-Villarreal et al. (2014). In this report we only focus on the fMRI part of the experiment. The order of patients' attendance to the behavioral and fMRI experiments was counter-balanced to control for any prior exposure confounds. The inclusion and exclusion criteria for participation in the fMRI experiment are described in Table 1. The patients' comorbidities and medications are described in Supplementary Table S1. The only male patient of the experimental sample was excluded due to an incidental radiological finding in the anatomical brain images. One female subject was excluded due to claustrophobia during the acquisition. Hence, a total of 20 females with a median age of 49 (22–70) were included for the final fMRI analysis in study. The study was approved by the Bioethics Committee of the Institute of Neurobiology, UNAM and informed written consents were obtained from all patients. The study was conducted in accordance with the Declaration of Helsinki and the patients received no compensation for taking part in the study, although transportation expenses (i.e., taxi) were covered if needed.

**Table 1:** **Patient selection criteria**.

**INCLUSION CRITERIA**
• Meeting the FM 1990 and 2010 criteria (Wolfe et al., 1990, 2010)
• FM diagnosed by a trained Rheumatologist
• Spontaneous, continuous and intense pain in daily life (VRS >5 average of a month)
• Right-handed
**EXCLUSION CRITERIA**
• Impossibility to move or walk
• Uncontrolled endocrine problems
• Auditory problems
• Pregnancy and/or breast-feeding
• MRI contraindications (i.e., metal prosthetics)
**ELIMINATION CRITERIA**
• Excessive MRI artifacts
• Probable pathological findings in MRI

*VRS, Verbal Rating Scale*.

### Design and paradigm

All patients filled out the Pain Catastrophizing Scale (PCS) and the Center for Epidemiologic Studies Depression Scale Revised (CESD-R) questionnaires prior to the experiment (Sullivan et al., 1995; Ortega et al., 2003). Pain intensity (PI) and Pain unpleasantness (PU) were measured using the 10-point Verbal Rating Scale (VRS) (0 = no pain, 10 = very intense/unpleasant pain) (Cork et al., 2004) immediately before and after each experimental condition in the MRI scanning. The experimental conditions consisted of two different auditory backgrounds that the patients listened to for 5 min each while the MRI scanner was not acquiring images: music (2–3 songs in a row) and pink noise (control). Prior to the study, the patients either provided the name of an artist or the songs they would like to hear, provided the musical pieces were highly pleasant and slow-paced. The slow pace was defined as a tempo of <120 beats per minute (bpm), determined by the main researcher using a metronome. Pleasantness was reported by the participant using a verbal 10-point Likert scale (0 = unpleasant, 10 = highly pleasant) and we defined pleasant music as rated at least 9–10 points. When only the artist name was provided, the experimenter chose the songs by the artist based on two fixed acoustic criteria: consonance (pleasantness), reported by the patient, and tempo, measures by the experimenter.

**Figure 1:**
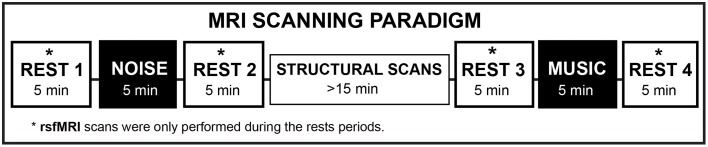
**Experimental rsfMRI paradigm**. The washout condition was executed during the “Structural Scans” period. NOISE, Control condition (pink noise). rsfMRI, resting state functional magnetic resonance imaging.

Figure 1 shows the experimental paradigm. For the fMRI experiment, the patients listened to the auditory stimuli inside the MRI scanner, during which period no sequences were running to minimize unwanted noise. The patients used the NordicNeuroLab AS (Bergen, Norway) MRI-safe headphones. The visual stimuli (fixation cross and wash-out) were presented in a screen projected through the mirror mounted on the MRI head coil. As compared to the behavioral study published in Garza-Villarreal et al. (2014), we added a wash-out condition during the structural imaging acquisition to avoid analgesic or cognitive crossing effects. The wash-out condition consisted of watching a neutral video documentary (i.e., a biography of Bill Gates) with sound. The documentary was rated as neutral by the researchers. All stimuli were presented using the software VLC Media Player (http://www.videolan.org). As shown in Figure 1, for a session there were four rsfMRI acquisitions in total (REST 1, REST 2, REST 3, and REST 4) with two auditory stimuli: Control (noise) and Music. A total of four conditions were then defined in the analysis: pre-control (Cpre), post-control (Cpos), and pre-music (Mpre), post-music (Mpos). The order of auditory stimulus presentation was counter-balanced across patients to avoid an order effect.

### Procedure

Patients were screened and recruited by the main researcher. During the screening, they were informed about the study, without being specific as to the tasks involved and the main objective (MIA). After the screening, the patients were first interviewed by phone to make sure they fit the inclusion criteria. After the patients were confirmed to be eligible to participate in the study, they were asked for songs they would like to listen during the study with the characteristics explained in the previous section. Before the scans they were briefed about the study, to make sure they understood the procedure and implications. The patients then signed the written consent, answered the questionnaires and were briefed about the details of the fMRI paradigm. During the fMRI scanning the patients rated their spontaneous pain before and after each auditory condition.

### Statistical analysis of pain measures

The VRS pain measures were analyzed using the software R Statistics (Team, 2014). We performed descriptive statistics of the data and plots using the “ggplot2” package for R (Wickham, 2009). PI and PU were not normally distributed; therefore, we chose to use non-parametric two-tailed paired analysis using the Mann–Whitney Rank Test. We performed this test in the difference variables ΔPI (pre–post PI) and ΔPU (pre–post PU) between the two experimental conditions: Control and Music. Three patients were excluded from this analysis due availability of the pain ratings data (*n* = 17).

### MRI data acquisition

The image acquisition was performed at the Magnetic Resonance Unit of the Institute of Neurobiology, UNAM, Queretaro, Mexico using a 3.0 Tesla GE Discovery MR750 scanner (HD, General Electric Healthcare, Waukesha, WI, USA) and a commercial 32-channel head coil array. High-resolution T1-weighted anatomical images were obtained using the FSPGR BRAVO pulse sequence: Plane orientation = Sagittal, TR = 7.7 ms, TE = 3.2 ms, flip angle = 12°, matrix = 256 × 256, FOV = 256 mm^2^, slice thickness = 1.1 mm, number of slices = 168, slice order = interleaved, view order = bottom-up. A gradient echo sequence was used to collect rsfMRI data using the following parameters: TR = 3000 ms, TE = 40 ms, flip angle = 90, matrix = 128 × 128, FOV = 256 mm^2^, slice thickness = 3 mm, voxel size = 2 × 2, slice spacing = 0 mm, plane orientation = axial, slice order = interleaved, view order = bottom-up, number of slices = 43. The total scan time of each rsfMRI session was 5 min with a total of 100 brain volumes acquired. During the rsfMRI the patients were given no task but were instructed to stay alert and keep their eyes open and fixated on a white-cross displayed on the center of black background that was being presented on the MRI screen. All images were downloaded in DICOM format, anonymized and converted to NIFTI format using dcm2nii from MRIcron (Rorden and Brett, 2000).

### Fractional amplitude of low frequency fluctuations (fALFF)

All image processing and data analysis of rsfMRI were performed using AFNI (http://afni.nimh.nih.gov/afni) (Cox, 1996) software and FMRIB's Software Libraries (FSL V5.0.4) (Smith et al., 2004; Woolrich et al., 2009; Jenkinson et al., 2012). We performed slice timing correction and motion correction. For each subject and session, after an initial rigid alignment between functional data and T1-weighted high-resolution structural images, a non-linear transformation field was obtained to register individual T1-weighted images to the Montreal Neurological Institute (MNI) standard space. Mean signal from white matter, cerebrospinal fluid, and the global signal were obtained from the T1-weighted images segmentation. Those signals and six motion parameters were regressed out from preprocessed images using linear regression. Finally we smoothed the residual images using a Gaussian kernel of full width at half maximum of 6 mm. All further image processing was carried out on the smoothed residual images. The main analysis of our resting state data was done using fALFF, an approach based on power density frequency spectrum (Zou et al., 2008). The fALFF was computed using the scripts released by 1000 Functional Connectomes Project (http://fcon_1000.projects.nitrc.org/). After Fisher-Z transformation, the individual fALFF maps were then analyzed with whole-brain two-sample paired *t*-tests using the toolbox “randomize” from FSL (Winkler et al., 2014) set at 5000 permutations to obtain differences in BOLD signal amplitude between all conditions. We performed a GLM analysis between PI and fALFF in all conditions, to find the neural correlates of PI report in this population. We then focused in the contrasts Mpos > Cpos and Mpos < Cpos because we were interested in the effect of the music compared to the control. However, we tested for differences between all conditions. To test for a possible effect of the normal fALFF fluctuation in time, we also tested REST 1 (baseline) against the rest of the REST sequences. Correction for multiple comparisons was performed with family-wise error (FWE) with an alpha level of 0.05 (Nichols and Hayasaka, 2003). We then extracted the mean fALFF Z-scores from each patient and session (Cpos and Mpos) using a binary mask ROI created from the group's significant cluster of the contrast Mpos > Cpos, and we performed parametric two-tailed correlations between the delta (Δ) Z-scores of the fALFF (Mpos–Cpos) and the ΔPI and PU (converted to Z-scores) using R Statistics (Team, 2014). Outliers of pain ratings were rejected using Thompson's Tau (Thompson, 1985; Christensen et al., 1992), with a final *n* = 15 for the correlation analysis. For clarity the following abbreviations will be used in the Results Section: Cpos, post-Control; Mpos, post-Music; Z, mean Z-score of the fALFF cluster; PI, pain intensity; PU, pain unpleasantness.

### Connectivity analysis

As a *post-hoc* analysis of the fALFF, in order to examine the functional connectivity of the significant cluster, we performed seed-based connectivity analysis. We band-passed the preprocessed MRI at 0.01–0.08 Hz, and then we extracted the mean fMRI time series of each subject using the mean from the angular gyrus cluster. Afterwards, we created individual correlation maps calculating the cross-correlations between a reference waveform (BOLD signal of the mean cluster) and time-series of each voxel in the whole-brain. To obtain the average correlations of conditions Cpos and Mpos, the individual correlation maps were Fisher's Z-transformed, averaged across all subjects for each condition, then transformed back to correlation coefficients. After mapping the positive and negative correlations of the lAnG for each condition at a chosen threshold of *r* = ±0.40, we decided to investigate the mean correlation of each cluster as an individual ROI. For this we clusterized Cpos and Mpos correlation maps with a positive lower threshold of *r* = 0.40, a negative lower threshold of *r* = −0.40, and a minimum cluster size of 40 voxels (except for the cluster located in left caudate (lCau) of 9 voxels in size, due to relevance in FM and pain literature). We then combined the Cpos and Mpos clusters to create the final ROIs. We ended up with nine ROIs from the positive correlations and 11 ROIs from the negative correlations with lAnG cluster (Figure S1). For clarity, we named the ROIs from the type of correlation they were derived from and a number in sequence (i.e., positive 1 or P1). Using these ROIs, we extracted the mean Z-value from the transformed correlation (*r*) maps from every individual subject for each condition (Cpos and Mpos) separately. Then, in the ROIs with a mean *r* difference (Cpos–Mpos) of > ± 0.10, we performed two-tailed parametric paired *t*-tests in the Z-values (Mpos > Cpos, Cpos > Mpos) using at alpha < 0.05, false-discovery rate (FDR) correction at *q* = 0.05 for multiple comparisons (Benjamini and Hochberg, 1995) and effect sizes were calculated as *r*^2^. Positive values after Mpos > Cpos are referred to as increased connectivity and negative values as decreases connectivity. Finally, to find about linear relationships between the pain reports and the lAnG connectivity, we created scatter plots of PI and the correlation coefficient (*r*) values of the significant ROIs, and we performed Pearson's two-tailed correlations of ROIs with linear relationship at alpha < 0.05. The correlation was done between the Δ PI (Mpos–Cpos) and the Δ Z-transformed correlation coefficient (*r*), reported afterwards in Pearson's *r*. All coordinates in the results are shown in MNI space.

## Results

### Questionnaires and behavioral pain measures

**Figure 2:**
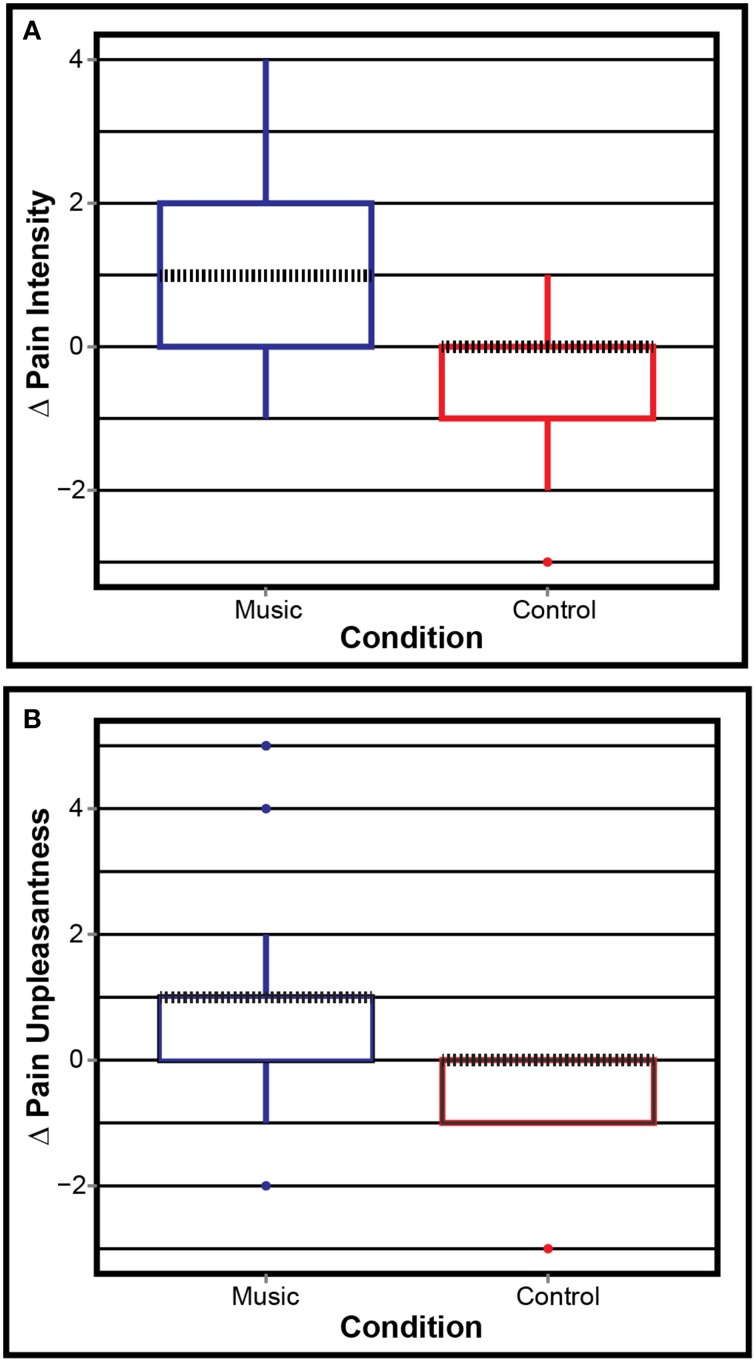
**Pain ratings**. **(A)** Pain intensity and **(B)** pain unpleasantness for both conditions. Positive values mean less pain and negative mean more pain after the condition. Δ, post–pre. Dotted black line shows the median.

The PCS (Mean/SD: 26.41/12.82) and CESD-R (Mean/SD: 25.36/11.95) results were as expected in this population: high pain catastrophizing and presence of depression symptoms in most patients. Figure 2 shows the boxplots of ΔPI and ΔPU on each condition. The Mann–Whitney paired test showed that Control and Music were significantly different in both ΔPI (*W* = 45, *p* = 0.008) and ΔPU (*W* = 96, *p* = 0.006). This suggests that the patients reported lower pain levels after listening to music, but not after listening to the control.

### Amplitude of low frequency fluctuations (fALFF)

**Figure 3:**
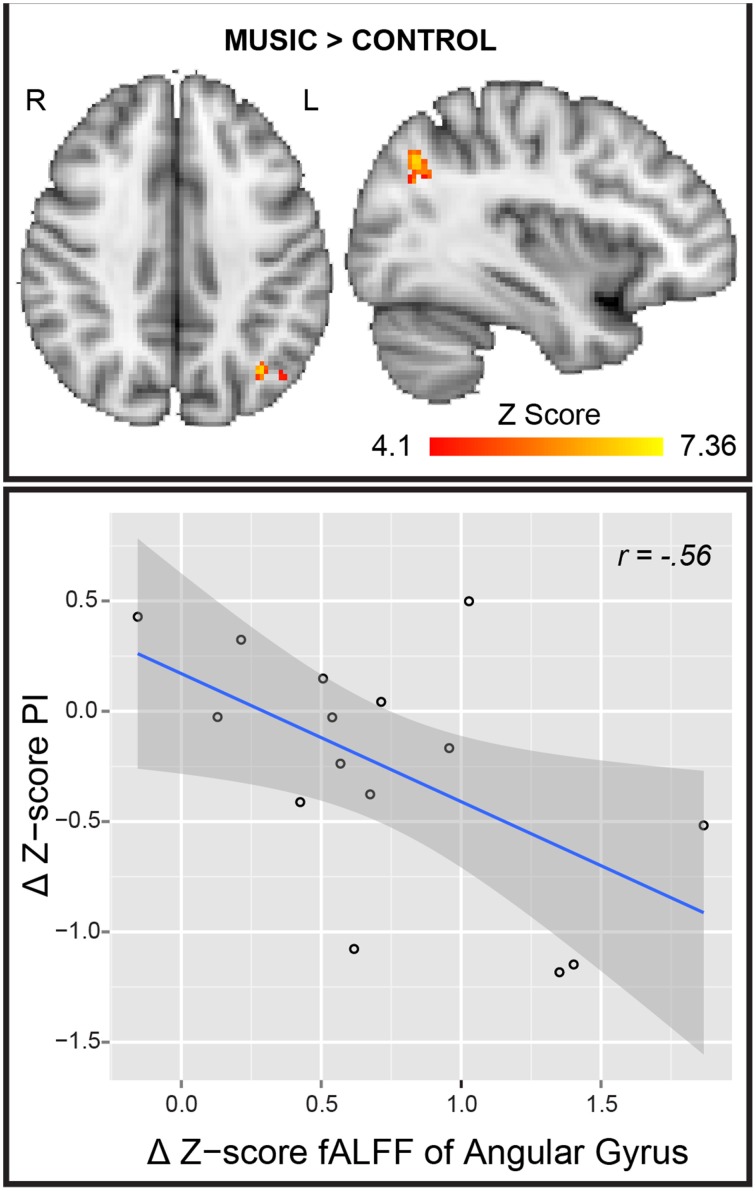
**fALFF Analysis. (Top)** Result of the fALFF Mpos > Cpos analysis. R, right; L, left. (**Bottom**) Scatter plot and regression line of Δ Z-score PI (y axis) and the Δ Z-score of the angular gyrus cluster. PI, pain intensity; *r*, Pearson's correlation coefficient; Mpos, post-Music; Cpos, post-Control; Δ, Mpos–Cpos.

The PI GLM analysis did not survive correction for multiple comparisons. However, at uncorrected *p* < 0.001 we found significant regression of the PI with fALFF in: bilateral posterior insula, left cerebellum and bilateral superior and medial temporal gyrus (Figure S2). The statistical analysis of Mpos > Cpos showed a significant cluster in the lAnG (center of mass, *x* = −38, *y* = −68, *z* = 36, *p* = 0.008, mean-*T* = 5.05, size = 64 voxels), meaning an increase in BOLD signal amplitude of the LFF after the music condition. No significant voxels were found in any other test. Furthermore, we found a significant negative correlation between Δ fALFF Z-score and Δ PI Z-score (*r* = −0.56, *p* = 0.03) (Figure 3). In other words, the patients who reported lower PI after the music showed higher BOLD amplitude in the lAnG.

### Connectivity of the left angular gyrus

**Figure 4:**
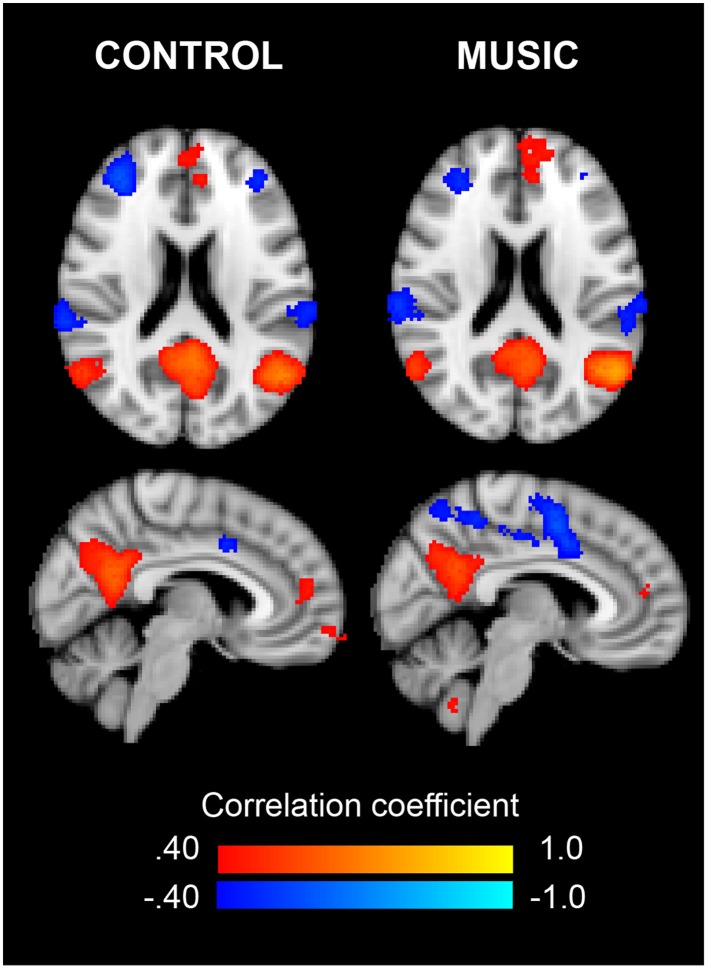
**Connectivity of the left angular gyrus for Control and Music**. Axial **(Top)** and sagittal **(Bottom)** views of the connectivity maps (threshold *r* = ±0.40) for the rsfMRI after the Control and after the Music, respectively. Colors show either positive (red–yellow) or negative (blue–light blue) correlations with the fALFF cluster in the left angular gyrus.

**Figure 5:**
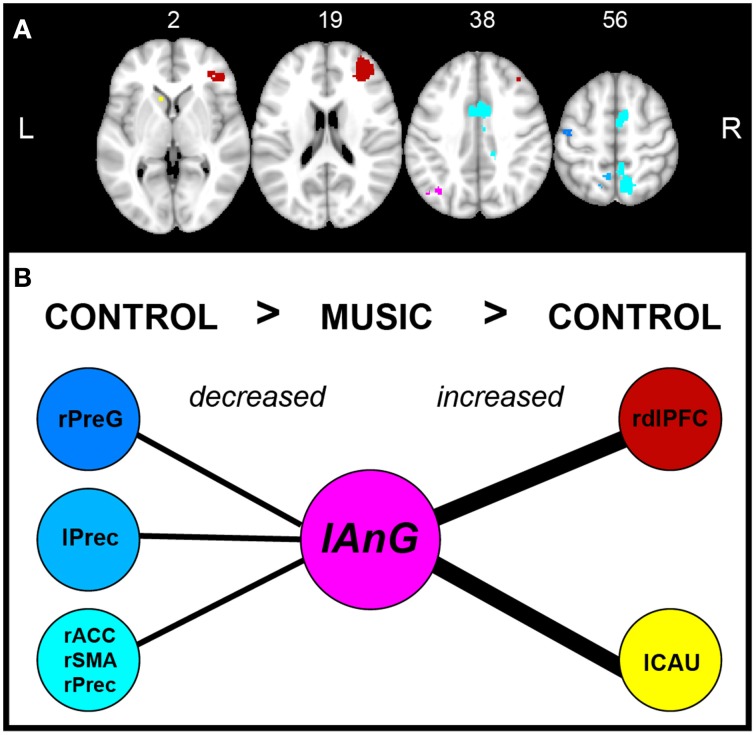
**Brain areas of connectivity with left angular gyrus. (A)** Axial view of the brain regions with increased or decreased connectivity with the lAnG (pink). Numbers on top show the slice location in the z-axis. Blue group represents the areas with decreased connectivity and Red group represents increased connectivity. The color intensity represents the *t*-values of the *t*-tests: blue (−1.93)—light blue (−3.44), and red (1.76)—yellow (2.58). **(B)** Schematic of the differences in connectivity of the lAnG with the other areas according to the statistical contrasts (post-Control > post-Music, and post-Music > post-Control). Thick lines represent increased connectivity and thin lines represent decreased connectivity. L, left; r, right; AnG, angular gyrus; PreG, Precentral gyrus; Prec, precuneus; ACC, anterior cingulate cortex; SMA, supplementary motor cortex; dlPFC, dorsolateral prefrontal cortex; CAU, caudate.

**Figure 6:**
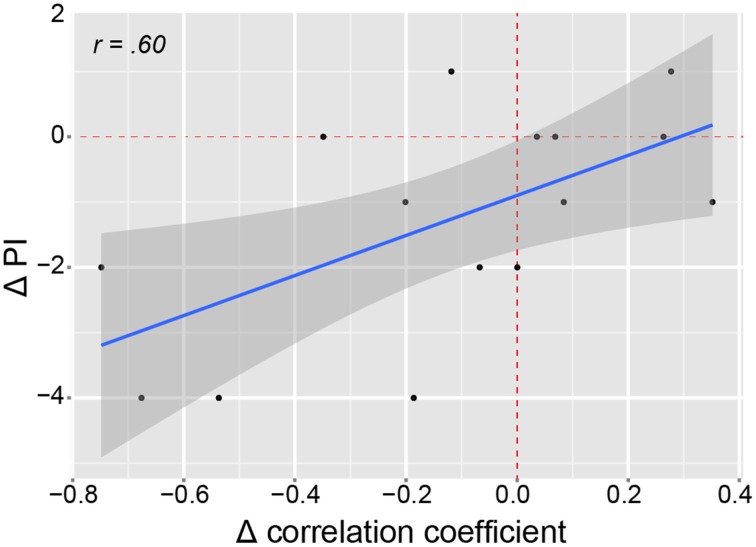
**Correlation of pain intensity and precentral-angular gyri connectivity**. Scatter plot showing the linear positive correlation between the Δ PI and the Δ correlation of the connectivity between lAnG and rPreG. Red dashed lines indicate vertical and horizontal lines passing the origin (0,0). The shaded area shows 95% confidence interval. Δ, delta (Mpos–Cpos); PI, pain intensity; *r*, correlation coefficient.

Figure 4 shows the correlation maps for Cpos and Mpos. In both conditions, the positive and negative correlations are related to the DMN (i.e., mPFC, IPL, precuneus, PCC), and other brain areas (i.e., ACC). In the statistical analysis, we found significant connectivity (correlation) differences in several ROIs, shown in Table 2 as increased or decreased connectivity with the lAnG (Figure 5). Finally, we found a significant positive correlation between Δ PI and Δ Z-values of the right precentral gyrus (rPreG) (*r* = 0.61, *p* = 0.02) (Figure 6).

**Table 2:** **Results of the paired ***t***-tests of the left angular gyrus connectivity with contrast post-Music > post-Control**.

**ROI**	**Region (s) of ROI**	**Mdiff. (r)**	**T**	***p*-Values**	***d***	**Connectivity**
P9	lCaudate	0.20	2.58	0.02^*^	0.45	Increased
N1	rACC-SMA-Precuneus	−0.21	−3.44	0.003^**^	0.46	Decreased
N2	lPrecuneus (BA 7)	−0.22	−2.57	0.02^*^	0.47	Decreased
N4	rPrecentral gyrus (BA 4)	−0.17	−1.93	0.09	0.41	Decreased
N7	rdlPFC (BA 9 and 10)	0.13	1.76	0.1	0.36	Increased

AnG, Angular gyrus; ROI, region of interest; Mdiff, mean of the differences (r); T, t-value; BA, Brodmann area; l, left; r, right; ACC, Anterior cingulate cortex; SMA, supplementary motor area; dlPFC, Dorsolateral prefrontal cortex. We show p-values passing FDR-correction:

* p < 0.05

** *p < 0.01, d = effect size (r^2^ of Mdiff.)*.

## Discussion

We investigated the resting state BOLD signal changes related to MIA in FM patients. We found that listening to pleasant, relaxing and familiar music reduced the patients' pain, and this was correlated with higher amplitude of the LFFs in the lAnG, an important part to the DMN. The connectivity analysis of the lAnG showed correlation with several areas of the DMN, with significant connectivity changes between conditions. Specifically, we found increased connectivity with the lCau, left dorsolateral prefrontal cortex (ldlPFC), and decreased connectivity with the right anterior cingulate cortex (rACC), right supplementary motor area (rSMA), bilateral precuneus and rPreG.

### Amplitude of low frequency fluctuations

Our rsfMRI results showed significantly higher amplitude of the LFFs in the lAnG after listening to music that was correlated with PI. The fALFF is a rather novel rsfMRI-based technique that aims at extracting further information from the LFFs, and the amplitude of the LFF is suggested to reflect the intensity of the spontaneous brain activity, which in turn seems to be related to physiological activity (Zou et al., 2008). It differs from connectivity analysis in that there are no measures of correlation between voxels; it solely informs about the amplitude of the spontaneous BOLD activity at a determined frequency band (0.01–0.08 Hz in this case), while correlation analysis is derived from the entire frequency bands. Therefore, even if correlations between brain areas do not change, the amplitude of their BOLD signal oscillations may change, suggesting a different type of “brain activation.” According to our results, there seems to be an association between the higher amplitude of the LFF observed in the lAnG and pain perception. This could mean that the activity in this core brain structure of the DMN may be influenced by the music, and in turn influence other areas to produce analgesia.

The angular gyrus is part of the inferior parietal lobule, it is an area involved in several cognitive domains (i.e., language processing, attention), and it is also an important node of the DMN (Greicius et al., 2008; Seghier, 2013). The DMN is known to be altered in different types of chronic pain diseases such as FM (Napadow et al., 2010). The AnG does not seem to be specifically dedicated to any cognitive domain, it is considered a functional association area, connecting with multiple brain areas and related to multiple cognitive functions, since fMRI studies demonstrated its involvement ranging from memory retrieval, attention, and mathematical processing (Uddin et al., 2010). Furthermore, it seems that the AnG is an important hub for multimodal integration, as it structurally connects with parietal, temporal, and frontal areas (Seghier, 2013).

BOLD activity of the AnG has been shown during high PI in task-based fMRI studies using experimental pain (Wiech et al., 2005) and during cognitive evaluation of the reported pain (Kong et al., 2006; Moulton et al., 2012). Another study showed activation of the lAnG in healthy participants related to somatosensory evaluation (Ushida et al., 2005), suggesting that the lAnG may be related to sensory evaluation of, but not specific to, pain. A recent task-based fMRI study in chronic pain patients showed increased activity in the lAnG following verbally induced placebo analgesia, but not following non-verbal cues (Craggs et al., 2014). Placebo analgesia is the analgesic effect of an innocuous agent, produced solely by the individual's belief of analgesia. Interestingly, the role of the AnG as a core region for integration of conceptual information has been described (Price et al., 2015). This suggests that in our study, the lAnG activity may have been related to a belief of “health” or analgesia from the music, verbal lyrics, previous memories and somatosensory information thereby suggesting that similar mechanisms may be involved in placebo and MIA. Cognitive evaluation of pain may also lead to reappraisal, a well-known mechanism of placebo analgesia consisting in the change or re-framing of the experience of pain (Wiech et al., 2008a; Tracey, 2010). Listening to self-chosen pleasant music may make the FM patients feel in control over the pain and elicit a reappraisal of the pain experience, producing analgesia. Nevertheless, the mere passive listening of music has also been shown to activate DMN brain areas (i.e., precuneus), suggesting influence of music in DMN connectivity (Alluri et al., 2012). Therefore, it is difficult to assume that the activity of the lAnG is closely related to the analgesic effect.

Another explanation about our result may relate to a domain in which the AnG has been consistently found to be involved in: reorienting or shifting attention (Singh-Curry and Husain, 2009; Seghier, 2013). Shifting attention or distraction from the pain is a well-known central (non-pharmacological) analgesia mechanism elicited by possibly a competition of cognitive resources (Wiech et al., 2008b). Inducing experimental acute pain in healthy participants, we previously found that an active distraction was an even stronger central analgesic than experimenter-chosen classical instrumental music (Garza-Villarreal et al., 2012). In our current study, the distraction from the pain by listening to self-chosen and pleasant music, containing lyrics, may have elicited an analgesic effect secondary to distraction that lingered even after the music ended (when we ran the rsfMRI acquisition) due to active memory recall, possibly linked to the memory processing function of the AnG. We need to stress that most of the music chosen by our patients contained lyrics. There is a difference in emotional brain processing of music between instrumental and lyrical music, as an fMRI study showed that sad music with lyrics is rated as more sad, and more strongly activates limbic system brain areas than sad instrumental music (Brattico et al., 2011). Other proposed mechanisms for MIA in our study may be positive emotions and relaxation, as they have been shown to reduce pain perception (Rhudy et al., 2008). However, emotional modulation of pain seems to be disrupted in FM (Kamping et al., 2013), hence we could not be certain of this mechanism. Either way, the correlation between the fALFF and PI further suggest that the changes in amplitude in the lAnG are somehow related to the MIA. To further understand the lAnG result, we performed a seed-based connectivity analysis of the significant fALFF cluster.

### Left angular gyrus connectivity

The mean correlation of the lAnG cluster in the post-Control and post-Music conditions showed a pattern of connectivity related to the DMN, suggesting that MIA was modulated by this higher-order network. The *post-hoc* connectivity analysis showed that the lAnG increased connectivity with the lCau, and right dorsolateral prefrontal cortex (rdlPFC); and decreased connectivity with the rACC, rSMA, precuneus and rPreG after the music. Although the rdlPFC and rPreG were not statistically significant, we decided to take them into consideration in this discussion due to the evidence of their involvement in pain and analgesia and their high effect sizes (>0.35 = medium – large effect).

Except for precuneus, these brain areas connected to the lAnG are part of the pain perception and modulatory network (Tracey and Dickenson, 2012), and found in most task and resting state fMRI pain studies. The prefrontal cortex is suggested to regulate pain perception in healthy subjects, represented by increased activity in either of its functional divisions, depending on the analgesic mechanism present (i.e., vlPFC, mPFC, and the dlPFC) (Lorenz et al., 2003; Tracey, 2011). In fact, areas that have been associated to a modulatory network in central (non-pharmacological) analgesia are: the rACC, OFC, and the dlPFC (Petrovic et al., 2005). The dlPFC has been related to central analgesia by reappraisal and distraction mechanisms (Wiech et al., 2008b). The ACC is part of the pain matrix and commonly found with increased activity during pain and decreased during analgesia (Tracey and Dickenson, 2012); hence it is considered to be related to the cognitive and emotional processing of pain (Kwan et al., 2000), as well as involved in arousal and attention (Paus, 2000). Interestingly, emotional modulation of pain seems to be affected in FM patients, reported as decreased task-based fMRI activation in S1, insula, OFC, and ACC during pain modulation by positive affect (Kamping et al., 2013). A study of FM resting state connectivity between FM patients and healthy participants found increased connectivity in FM patients of the ACC with the caudate, putamen and insula; the caudate with mPFC and secondary somatosensory cortex (S2); and the SMA with primary M1. Also, they found decreased connectivity of the ACC with the PAG and amygdala; caudate with PAG and GP (Cifre et al., 2012). In our results, we found the rACC, SMA, M1, and caudate being modulated by (or modulating) the lAnG, suggesting a top-down pain modulation by the DMN, possibly orchestrated by the AnG.

There are several studies in healthy subjects showing the neural correlates and connectivity during central analgesia (Wiech et al., 2008a; Petrovic et al., 2010; Hashmi et al., 2012). However, in FM literature there is only one study of rsfMRI brain connectivity during central analgesia (real and sham acupuncture) and they found decreased connectivity of the DMN to the anterior insula related to analgesia. In our study, we focused on the amplitude of the LFFs and not the DMN connectivity, and we did not find any insula changes. Interestingly, the lAnG connectivity changes were related to areas involved in pain and analgesia already shown in other studies. A meta-analysis of experimental pain and placebo analgesia showed that there is an overlap of areas related to pain and analgesia, suggesting instead that placebo analgesia may not arise from these areas themselves, but as a function of the activity of this pain modulatory network (Amanzio et al., 2013). In our results, the area with the highest effect size in increased connectivity was the lCau, and the areas with the highest effect size in decreased connectivity were the rACC, rSMA and rPrecuneus. However only the connectivity of lAnG with rPreG was correlated to PI changes.

It is therefore possible that in this experiment the lAnG was, for example, influencing (or being influenced) frontal hubs such as dlPFC, rACC, and SMA to decrease pain perception, and receiving feedback from areas such as lCau and PreG. PreG contains the primary M1, which has already been shown to have increased connectivity with the SMA in FM patients (Cifre et al., 2012). The precuneus is related to pain perception in pathologies with higher levels of psychological distress (similar to FM) and to altered cognitive states such as hypnosis (Albuquerque et al., 2006; Huber et al., 2013), therefore our connectivity results may be more related to relaxation than analgesia. It is important to remember that FM pain is not yet fully understood and this is also true for the neural representation of pain and central analgesia in FM. Studies suggest that FM patients have a disruption of pain inhibition areas such as the ACC (Jensen et al., 2009; Kamping et al., 2013). If this is the case, then the ability to modulate pain is impaired in FM patients and different pathways may compensate for this impairment. Here, we showed that FM patients can have an analgesic effect by listening music, and that this effect seems to be related to top–down mechanisms.

### Limitations

As with most FM patients, the patients of this study were under several types of medication and had several comorbidities that we could not control for. However, by doing paired design (within-subjects) we tried to control for this individual variability. It should also be noted that most patients showed depression symptoms, a common problem in FM. In this case it was not possible to control this variable and, for future studies, it would be important to understand which of the brain correlates we found are secondary to analgesia and which to a reduction of depression symptoms secondary to music exposure. The voxel size (2 × 2 × 3) may not show the activity of subcortical structures important for pain modulation (i.e., PAG), and the relatively low sampling rate (TR = 3 s) could overlook part of the temporal dynamics of the connectivity. A control group with healthy participants would have been ideal to possibly understand the effect of music in the lAnG. However, the groups could not be directly comparable as the healthy controls would have to be experiencing similar generalized intense pain without FM to be an ideal control group. Also, in healthy controls the goal of choosing music would serve a different purpose than the FM group. Finally, as we mentioned before, the FM brain pathology and variability make it difficult to compare and interpret results against other populations. Therefore, a future study should improve these technical limitations and sample size to better understand central analgesia in FM.

## Conclusions

Here we found that listening to pleasant, familiar and relaxing music reduces pain in FM, and that this MIA was related to increased amplitude of the low-frequency BOLD signal fluctuations in the lAnG and changes in connectivity between brain areas related to pain and analgesia. We propose that the analgesic effect in this study is a consequence of top-down mechanisms, by either placebo analgesia, distraction, positive emotions, or a combination of these mechanisms.

### Conflict of interest statement

The authors declare that the research was conducted in the absence of any commercial or financial relationships that could be construed as a potential conflict of interest.

## Supplementary material

Figure S1 **Sagittal view of the regions of interest chosen from the merge of Cpos and Mpos clusters of the whole brain correlations of the lAnG at a threshold *r* = ± 0.40**. Red represents the ROIs derived from clusters with positive correlation and blue are the clusters with negative correlation. Each ROI was named with a P (positive) or N (negative) and a number. X, x-axis; lAnG, left angular gyrus.

Figure S2 **Sagittal view of the Pain Intensity—fALFF BOLD signal regression clusters on right insula at uncorrected *p* < 0.001 (slice at x-axis = 38, in MNI space)**.

## References

[B1] Albuquerque R. J. C. de Leeuw R. Carlson C. R. Okeson J. P. Miller C. S. Andersen A. H. (2006). Cerebral activation during thermal stimulation of patients who have burning mouth disorder: an fMRI study. Pain 122, 223–234. 10.1016/j.pain.2006.01.020 16632202

[B2] Alluri V. Toiviainen P. Jääskeläinen I. P. Glerean E. Sams M. Brattico E. (2012). Large-scale brain networks emerge from dynamic processing of musical timbre, key and rhythm. Neuroimage 59, 3677–3689. 10.1016/j.neuroimage.2011.11.019 22116038

[B3] Amanzio M. Benedetti F. Porro C. A. Palermo S. Cauda F. (2013). Activation likelihood estimation meta-analysis of brain correlates of placebo analgesia in human experimental pain. Hum. Brain Mapp. 34, 738–752. 10.1002/hbm.21471 22125184 PMC6870130

[B4] Benjamini Y. Hochberg Y. (1995). Controlling the false discovery rate: a practical and powerful approach to multiple testing. J. R. Stat. Soc. B 57, 289–300.

[B5] Bingel U. Tracey I. (2008). Imaging CNS modulation of pain in humans. Physiology 23, 371–380. 10.1152/physiol.00024.2008 19074744

[B6] Biswal B. B. Mennes M. Zuo X.-N. Gohel S. Kelly C. Smith S. M.. (2010). Toward discovery science of human brain function. Proc. Natl. Acad. Sci. U.S.A. 107, 4734–4739. 10.1073/pnas.0911855107 20176931 PMC2842060

[B7] Brattico E. Alluri V. Bogert B. Jacobsen T. Vartiainen N. Nieminen S.. (2011). A functional MRI study of happy and sad emotions in music with and without lyrics. Front. Psychol. 2:308. 10.3389/fpsyg.2011.00308 22144968 PMC3227856

[B8] Brederson J.-D. Jarvis M. F. Honore P. Surowy C. S. (2011). Fibromyalgia: mechanisms, current treatment and animal models. Curr. Pharm. Biotechnol. 12, 1613–1626. 10.2174/138920111798357258 21466451

[B9] Christensen R. Pearson L. M. Johnson W. (1992). Case-deletion diagnostics for mixed models. Technometrics. 34, 38–45. 10.1080/00401706.1992.10485231

[B10] Cifre I. Sitges C. Fraiman D. Muñoz M. Á. Balenzuela P. González-Roldán A.. (2012). Disrupted functional connectivity of the pain network in fibromyalgia. Psychosom. Med. 74, 55–62. 10.1097/PSY.0b013e3182408f04 22210242

[B11] Clauw D. J. (2009). Fibromyalgia: an overview. Am. J. Med. 122, S3–S13. 10.1016/j.amjmed.2009.09.006 19962494

[B12] Cork R. C. Isaac I. Eisharydah A. Saleemi S. Zavisca F. Alexander L. (2004). A comparison of the verbal rating scale and the visual analog scale for pain assessment. Internet J. Anesthesiol. 8 10.5580/1a73

[B13] Cox R. W. (1996). AFNI: software for analysis and visualization of functional magnetic resonance neuroimages. Comput. Biomed. Res. 29, 162–173 8812068 10.1006/cbmr.1996.0014

[B14] Craggs J. G. Price D. D. Robinson M. E. (2014). Enhancing the placebo response: functional magnetic resonance imaging evidence of memory and semantic processing in placebo analgesia. J. Pain 15, 435–446. 10.1016/j.jpain.2013.12.009 24412799 PMC4004374

[B15] Finlay K. A. (2014). Music-induced analgesia in chronic pain: efficacy and assessment through a primary-task paradigm. Psychol. Music 42, 325–346. 10.1177/0305735612471236

[B16] Garza-Villarreal E. A. Brattico E. Vase L. Ostergaard L. Vuust P. (2012). Superior analgesic effect of an active distraction versus pleasant unfamiliar sounds and music: the influence of emotion and cognitive style. PLoS ONE 7:e29397. 10.1371/journal.pone.0029397.t003 22242169 PMC3252324

[B17] Garza-Villarreal E. A. Wilson A. D. Vase L. Brattico E. Barrios F. A. Jensen T. S.. (2014). Music reduces pain and increases functional mobility in fibromyalgia. Front. Psychol. 5:90. 10.3389/fpsyg.2014.00090 24575066 PMC3920463

[B18] Geisser M. E. Gracely R. H. Giesecke T. Petzke F. W. Williams D. A. Clauw D. J. (2007). The association between experimental and clinical pain measures among persons with fibromyalgia and chronic fatigue syndrome. Eur. J. Pain 11, 202–207. 10.1016/j.ejpain.2006.02.001 16546424

[B19] Greicius M. D. Krasnow B. Reiss A. L. Menon V. (2003). Functional connectivity in the resting brain: a network analysis of the default mode hypothesis. Proc. Natl. Acad. Sci. U.S.A. 100, 253–258. 10.1073/pnas.0135058100 12506194 PMC140943

[B20] Greicius M. D. Supekar K. Menon V. Dougherty R. F. (2008). Resting-state functional connectivity reflects structural connectivity in the default mode network. Cereb. Cortex 19, 72–78. 10.1093/cercor/bhn059 18403396 PMC2605172

[B21] Guétin S. Giniès P. Siou D. K. A. Picot M.-C. Pommié C. Guldner E.. (2012). The effects of music intervention in the management of chronic pain: a single-blind, randomized, controlled trial. Clin. J. Pain 28, 329–337. 10.1097/AJP.0b013e31822be973 22001666

[B22] Gutgsell K. J. Schluchter M. Margevicius S. DeGolia P. A. McLaughlin B. Harris M.. (2013). Music therapy reduces pain in palliative care patients: a randomized controlled trial. J. Pain Symptom Manag. 45, 822–831. 10.1016/j.jpainsymman.2012.05.008 23017609

[B23] Hashmi J. A. Baria A. T. Baliki M. N. Huang L. Schnitzer T. J. Apkarian A. V. (2012). Brain networks predicting placebo analgesia in a clinical trial for chronic back pain. Pain 153, 2393–2402. 10.1016/j.pain.2012.08.008 22985900 PMC3494789

[B24] Hong J.-Y. Kilpatrick L. A. Labus J. Gupta A. Jiang Z. Ashe-McNalley C.. (2013). Patients with chronic visceral pain show sex-related alterations in intrinsic oscillations of the resting brain. J. Neurosci. 33, 11994–12002. 10.1523/JNEUROSCI.5733-12.2013 23864686 PMC3713732

[B25] Hsieh C. Kong J. Kirsch I. Edwards R. R. Jensen K. B. Kaptchuk T. J.. (2014). Well-loved music robustly relieves pain: a randomized, controlled trial. PLoS ONE 9:e107390. 10.1371/journal.pone.0107390.s003 25211164 PMC4161415

[B26] Huber A. Lui F. Porro C. A. (2013). Hypnotic susceptibility modulates brain activity related to experimental placebo analgesia. Pain 154, 1509–1518. 10.1016/j.pain.2013.03.031 23664683

[B27] Jenkinson M. Beckmann C. F. Behrens T. E. J. Woolrich M. W. Smith S. M. (2012). FSL. Neuroimage 62, 782–790. 10.1016/j.neuroimage.2011.09.015 21979382

[B28] Jensen K. B. Kosek E. Petzke F. Carville S. Fransson P. Marcus H.. (2009). Evidence of dysfunctional pain inhibition in Fibromyalgia reflected in rACC during provoked pain. Pain 144, 95–100. 10.1016/j.pain.2009.03.018 19410366

[B29] Jensen K. B. Loitoile R. Kosek E. Petzke F. Carville S. Fransson P.. (2012). Patients with fibromyalgia display less functional connectivity in the brain's pain inhibitory network. Mol. Pain 8:32. 10.1186/1744-8069-8-32 22537768 PMC3404927

[B30] Jorge L. L. Amaro E. (2012). Brain imaging in fibromyalgia. Curr. Pain Headache Rep. 16, 388–398. 10.1007/s11916-012-0284-9 22717698

[B31] Kamping S. Bomba I. C. Kanske P. Diesch E. Flor H., (2013). Deficient modulation of pain by a positive emotional context in fibromyalgia patients. Pain 154, 1846–1855. 10.1016/j.pain.2013.06.003 23752177

[B32] Kim J.-Y. Kim S.-H. Seo J. Kim S.-H. Han S. W. Nam E. J.. (2013a). Increased power spectral density in resting-state pain-related brain networks in fibromyalgia. Pain 154, 1792–1797. 10.1016/j.pain.2013.05.040 23714266

[B33] Kim S.-H. Lee Y. Lee S. Mun C.-W. (2013b). Evaluation of the effectiveness of pregabalin in alleviating pain associated with fibromyalgia: using functional magnetic resonance imaging study. PLoS ONE 8:e74099. 10.1371/journal.pone.0074099.g008 24040178 PMC3765321

[B34] Kong J. White N. S. Kwong K. K. Vangel M. G. Rosman I. S. Gracely R. H.. (2006). Using fMRI to dissociate sensory encoding from cognitive evaluation of heat pain intensity. Hum. Brain Mapp. 27, 715–721. 10.1002/hbm.20213 16342273 PMC6871429

[B35] Kwan C. L. Crawley A. P. Mikulis D. J. Davis K. D. (2000). An fMRI study of the anterior cingulate cortex and surrounding medial wall activations evoked by noxious cutaneous heat and cold stimuli. Pain 85, 359–374. 10.1016/S0304-3959(99)00287-0 10781909

[B36] Lorenz J. Minoshima S. Casey K. L. (2003). Keeping pain out of mind: the role of the dorsolateral prefrontal cortex in pain modulation. Brain 126, 1079–1091. 10.1093/brain/awg102 12690048

[B37] Matsota P. Christodoulopoulou T. Smyrnioti M. E. Pandazi A. Kanellopoulos I. Koursoumi E.. (2013). Music's use for anesthesia and analgesia. J. Altern. Complement. Med. 19, 298–307. 10.1089/acm.2010.0235 22989077

[B38] McCaffrey R. Freeman E. (2003). Effect of music on chronic osteoarthritis pain in older people. J. Adv. Nurs. 44, 517–524. 10.1046/j.0309-2402.2003.02835.x 14651700

[B39] Mitchell L. A. MacDonald R. A. R. Brodie E. (2006). A comparison of the effects of preferred music, arithmetic and humour on cold pressor pain. Eur. J. Pain 10, 343–351. 10.1016/j.ejpain.2005.03.005 15878297

[B40] Mitchell L. A. MacDonald R. A. (2006). An experimental investigation of the effects of preferred and relaxing music listening on pain perception. J. Music Ther. 43, 295–316. 10.1093/jmt/43.4.295 17348757

[B41] Mitchell L. A. MacDonald R. A. R. Knussen C. Serpell M. G. (2007). A survey investigation of the effects of music listening on chronic pain. Psychol. Music 35, 37–57. 10.1177/0305735607068887

[B42] Moulton E. A. Pendse G. Becerra L. R. Borsook D. (2012). BOLD responses in somatosensory cortices better reflect heat sensation than pain. J. Neurosci. 32, 6024–6031. 10.1523/JNEUROSCI.0006-12.2012 22539862 PMC3347471

[B43] Napadow V. Kim J. Clauw D. J. Harris R. E. (2012). Brief report: decreased intrinsic brain connectivity is associated with reduced clinical pain in fibromyalgia. Arthritis Rheum. 64, 2398–2403. 10.1002/art.34412 22294427 PMC3349799

[B44] Napadow V. LaCount L. Park K. As-Sanie S. Clauw D. J. Harris R. E. (2010). Intrinsic brain connectivity in fibromyalgia is associated with chronic pain intensity. Arthritis Rheum. 62, 2545–2555. 10.1002/art.27497 20506181 PMC2921024

[B45] Nebel M. B. Gracely R. H. (2009). Neuroimaging of fibromyalgia. Rheum. Dis. Clin. North Am. 35, 313–327. 10.1016/j.rdc.2009.06.004 19647145

[B46] Nichols T. Hayasaka S. (2003). Controlling the familywise error rate in functional neuroimaging: a comparative review. Stat. Methods Med. Res. 12, 419–446. 10.1191/0962280203sm341ra 14599004

[B47] Onieva-Zafra M. D. Castro-Sánchez A. M. Matarán-Peñarrocha G. A. Moreno-Lorenzo C. (2013). Effect of music as nursing intervention for people diagnosed with fibromyalgia. Pain Manag. Nurs. 14, e39–e46. 10.1016/j.pmn.2010.09.004 23108015

[B48] Ortega M. R. Hernández A. L. S. Kegel J. G. M. (2003). Actualización de la escala de depresión del dentro de estudios epidemiológicos (CES-D). Estudio piloto en una muestra geriátrica mexicana. Salud Ment. 26, 59–68.

[B49] Özer N. Karaman Özlü Z. Arslan S. Günes N. (2013). Effect of music on postoperative pain and physiologic parameters of patients after open heart surgery. Pain Manag. Nurs. 14, 20–28. 10.1016/j.pmn.2010.05.002 23452523

[B50] Paus T. (2000). Functional anatomy of arousal and attention systems in the human brain. Prog. Brain Res. 126, 65–77. 10.1016/S0079-6123(00)26007-X 11105640

[B51] Pereira C. S. Teixeira J. Figueiredo P. Xavier J. Castro S. L. Brattico E. (2011). Music and emotions in the brain: familiarity matters. PLoS ONE 6:e27241. 10.1371/journal.pone.0027241 22110619 PMC3217963

[B52] Petrovic P. Dietrich T. Fransson P. Andersson J. Carlsson K. Ingvar M. (2005). Placebo in emotional processing—induced expectations of anxiety relief activate a generalized modulatory network. Neuron 46, 957–969. 10.1016/j.neuron.2005.05.023 15953423

[B53] Petrovic P. Kalso E. Petersson K. M. Andersson J. Fransson P. Ingvar M. (2010). A prefrontal non-opioid mechanism in placebo analgesia. Pain 150, 59–65. 10.1016/j.pain.2010.03.011 20399560

[B54] Price A. R. Bonner M. F. Peelle J. E. Grossman M. (2015). Converging evidence for the neuroanatomic basis of combinatorial semantics in the angular gyrus. J. Neurosci. 35, 3276–3284. 10.1523/JNEUROSCI.3446-14.2015 25698762 PMC4331639

[B55] Pujol J. Macià D. Garcia-Fontanals A. Blanco-Hinojo L. López-Solà M. Garcia-Blanco S.. (2014). The contribution of sensory system functional connectivity reduction to clinical pain in fibromyalgia. Pain 155, 1492–1503. 10.1016/j.pain.2014.04.028 24792477

[B56] Rhudy J. L. Williams A. E. McCabe K. M. Russell J. L. Maynard L. J. (2008). Emotional control of nociceptive reactions (ECON): do affective valence and arousal play a role? Pain 136, 250–261. 10.1016/j.pain.2007.06.031 17703886

[B57] Rorden C. Brett M. (2000). Stereotaxic display of brain lesions. Behav. Neurol. 12, 191–200. 10.1155/2000/421719 11568431

[B58] Roy M. Lebuis A. Hugueville L. Peretz I. Rainville P. (2012). Spinal modulation of nociception by music. Eur. J. Pain 16, 870–877. 10.1002/j.1532-2149.2011.00030.x 22337476

[B59] Roy M. Peretz I. Rainville P. (2008). Emotional valence contributes to music-induced analgesia. Pain 134, 140–147. 10.1016/j.pain.2007.04.003 17532141

[B60] Salimpoor V. N. van den Bosch I. Kovacevic N. McIntosh A. R. Dagher A. Zatorre R. J. (2013). Interactions between the nucleus accumbens and auditory cortices predict music reward value. Science 340, 216–219. 10.1126/science.1231059 23580531

[B61] Scott D. J. Stohler C. S. Egnatuk C. M. Wang H. Koeppe R. A. Zubieta J.-K. (2007). Individual differences in reward responding explain placebo-induced expectations and effects. Neuron 55, 325–336. 10.1016/j.neuron.2007.06.028 17640532

[B62] Seghier M. L. (2013). The angular gyrus: multiple functions and multiple subdivisions. Neuroscientist 19, 43–61. 10.1177/1073858412440596 22547530 PMC4107834

[B63] Singh-Curry V. Husain M. (2009). The functional role of the inferior parietal lobe in the dorsal and ventral stream dichotomy. Neuropsychologia 47, 1434–1448. 10.1016/j.neuropsychologia.2008.11.033 19138694 PMC2697316

[B64] Smith H. S. Harris R. Clauw D. (2011). Fibromyalgia: an afferent processing disorder leading to a complex pain generalized syndrome. Pain Physician 14, E217–E245. 10.1016/b978-1-4377-2242-0.00058-4 21412381

[B65] Smith S. M. Jenkinson M. Woolrich M. W. Beckmann C. F. Behrens T. E. J. Johansen-Berg H.. (2004). Advances in functional and structural MR image analysis and implementation as FSL. Neuroimage 23(Suppl. 1), S208–S219. 10.1016/j.neuroimage.2004.07.051 15501092

[B66] Sullivan M. J. L. Bishop S. R. Pivik J. (1995). The pain catastrophizing scale: development and validation. Psychol. Assess. 7, 524–532

[B67] Team R. C. (2014). R: A Language and Environment for Statistical Computing. Available online at: http://www.R-project.org

[B68] Thompson R. (1985). A note on restricted maximum likelihood estimation with an alternative outlier model. J. R. Stat. Soc. Ser. B. 47, 53–55.

[B69] Tracey I. (2010). Getting the pain you expect: mechanisms of placebo, nocebo and reappraisal effects in humans. Nat. Med. 16, 1277–1283. 10.1038/nm.2229 20948533

[B70] Tracey I. Dickenson A. (2012). SnapShot: pain perception. Cell 148, 1308–1308.e2. 10.1016/j.cell.2012.03.004 22424237

[B71] Tracey I. (2011). Can neuroimaging studies identify pain endophenotypes in humans? Nat. Rev. Neurol. 7, 173–181. 10.1038/nrneurol.2011.4 21304481

[B72] Uddin L. Q. Supekar K. Amin H. Rykhlevskaia E. Nguyen D. A. Greicius M. D.. (2010). Dissociable connectivity within human angular gyrus and intraparietal sulcus: evidence from functional and structural connectivity. Cereb. Cortex 20, 2636–2646. 10.1093/cercor/bhq011 20154013 PMC2951845

[B73] Ushida T. Ikemoto T. Taniguchi S. Ishida K. Murata Y. Ueda W.. (2005). Virtual pain stimulation of allodynia patients activates cortical representation of pain and emotions: a functional MRI study. Brain Topogr. 18, 27–35. 10.1007/s10548-005-7898-8 16193264

[B74] van den Bosch I. Salimpoor V. N. Zatorre R. J. (2013). Familiarity mediates the relationship between emotional arousal and pleasure during music listening. Front. Hum. Neurosci. 7:534. 10.3389/fnhum.2013.00534 24046738 PMC3763198

[B75] Wickham H. (2009). ggplot2: Elegant Graphics for Data Analysis. New York, NY: Springer Science & Business Media.

[B76] Wiech K. Farias M. Kahane G. Shackel N. Tiede W. Tracey I. (2008a). An fMRI study measuring analgesia enhanced by religion as a belief system. Pain 139, 467–476. 10.1016/j.pain.2008.07.030 18774224

[B77] Wiech K. Ploner M. Tracey I. (2008b). Neurocognitive aspects of pain perception. Trends Cogn. Sci. 12, 306–313. 10.1016/j.tics.2008.05.005 18606561

[B78] Wiech K. Seymour B. Kalisch R. Stephan K. E. Koltzenburg M. Driver J.. (2005). Modulation of pain processing in hyperalgesia by cognitive demand. Neuroimage 27, 59–69. 10.1016/j.neuroimage.2005.03.044 15978845

[B79] Winkler A. M. Ridgway G. R. Webster M. A. Smith S. M. Nichols T. E. (2014). Permutation inference for the general linear model. Neuroimage 92, 381–397. 10.1016/j.neuroimage.2014.01.060 24530839 PMC4010955

[B80] Wolfe F. Brähler E. Hinz A. Häuser W. (2013). Fibromyalgia prevalence, somatic symptom reporting, and the dimensionality of polysymptomatic distress: results from a survey of the general population. Arthritis Care Res. 65, 777–785. 10.1002/acr.21931 23424058

[B81] Wolfe F. Clauw D. J. Fitzcharles M.-A. Goldenberg D. L. Katz R. S. Mease P.. (2010). The American College of rheumatology preliminary diagnostic criteria for fibromyalgia and measurement of symptom severity. Arthritis Care Res. 62, 600–610. 10.1002/acr.20140 20461783

[B82] Wolfe F. Smythe H. A. Yunus M. B. Bennett R. M. Bombardier C. Goldenberg D. L.. (1990). The American College of rheumatology 1990 criteria for the classification of fibromyalgia. Arthritis and Rheum. 33, 160–172. 10.1002/art.1780330203 2306288

[B83] Woolrich M. W. Jbabdi S. Patenaude B. Chappell M. Makni S. Behrens T.. (2009). Bayesian analysis of neuroimaging data in FSL. Neuroimage 45, S173–S186. 10.1016/j.neuroimage.2008.10.055 19059349

[B84] Yeo J. K. Cho D. Y. Oh M. M. Park S. S. Park M. G. (2013). Listening to music during cystoscopy decreases anxiety, pain, and dissatisfaction in patients: a pilot randomized controlled trial. J. Endourol. 27, 459–462. 10.1089/end.2012.0222 23009573 PMC3624627

[B85] Zou Q.-H. Zhu C.-Z. Yang Y. Zuo X.-N. Long X.-Y. Cao Q.-J.. (2008). An improved approach to detection of amplitude of low-frequency fluctuation (ALFF) for resting-state fMRI: fractional ALFF. J. Neurosci. Methods 172, 137–141. 10.1016/j.jneumeth.2008.04.012 18501969 PMC3902859

