# Supplementary figures and images for: Music reduces pain and increases resting state fMRI BOLD signal amplitude in the left angular gyrus in fibromyalgia patients
Source: Front Psychol. 2015 Jul 22;6:1051. doi: 10.3389/fpsyg.2015.01051 (PMC4510313; doi:10.3389/fpsyg.2015.01051)

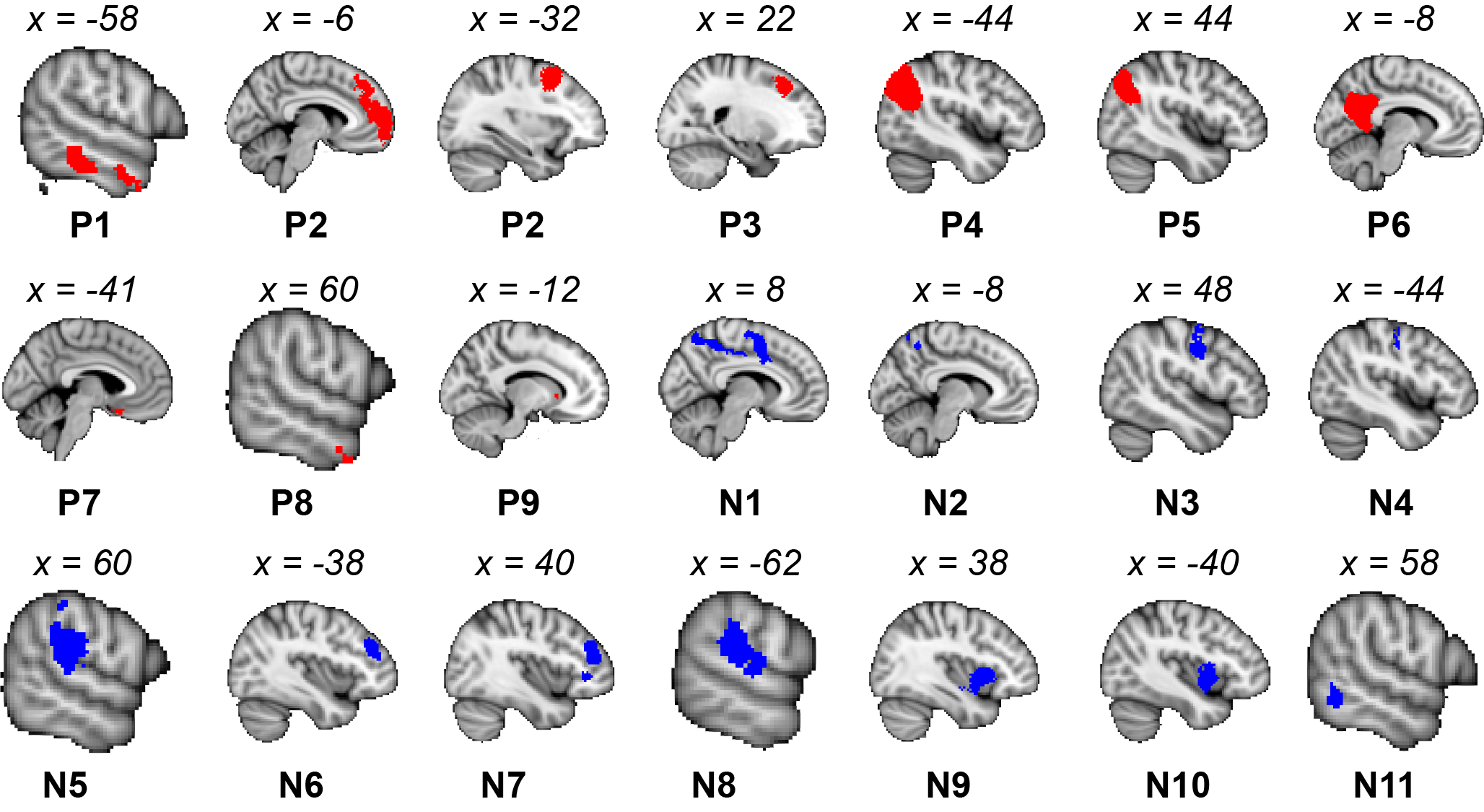

Supplement: Figure S1 — Sagittal view of the regions of interest chosen from the merge of Cpos and Mpos clusters of the whole brain correlations of the lAnG at a threshold r = ± 0.40. Red represents the ROIs derived from clusters with positive correlation and blue are the clusters with negative correlation. Each ROI was named with a P (positive) or N (negative) and a number. X, x-axis; lAnG, left angular gyrus. [file Image1.TIF]

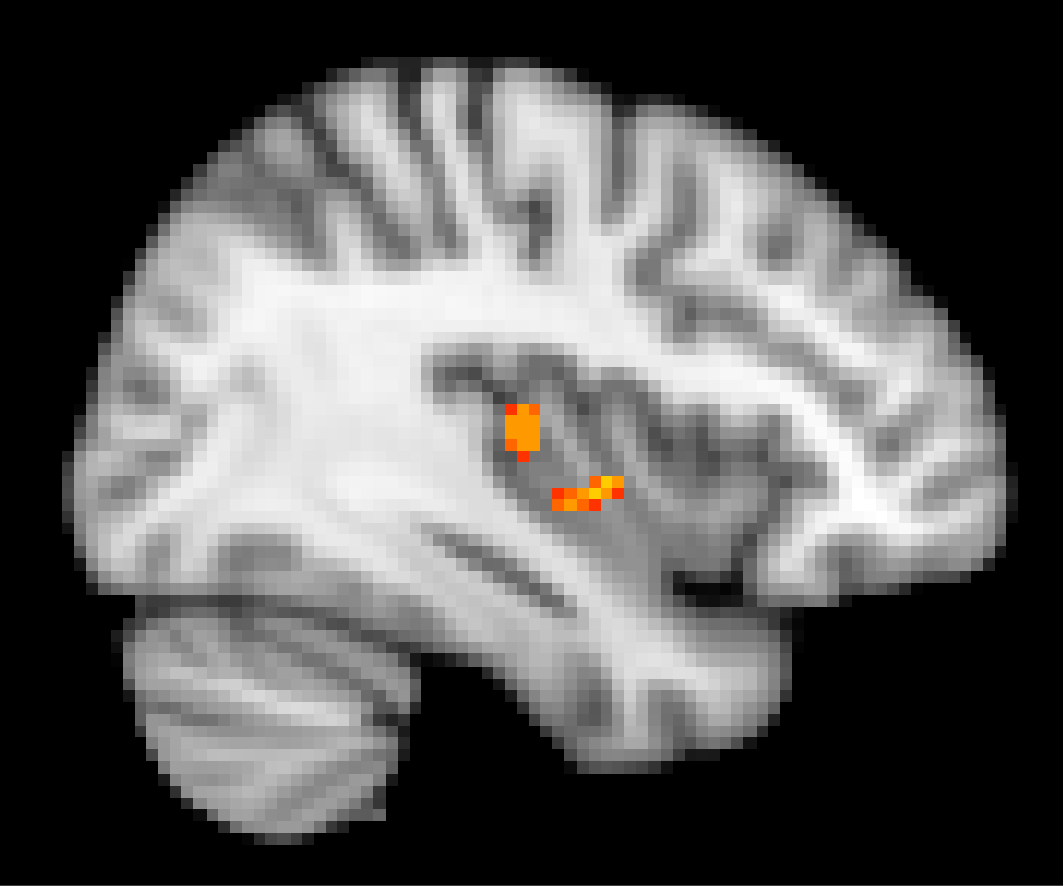

Supplement: Figure S2 — Sagittal view of the Pain Intensity—fALFF BOLD signal regression clusters on right insula at uncorrected p < 0.001 (slice at x-axis = 38, in MNI space). [file Image2.TIF]
