# Supplementary material for: Music reduces pain and increases resting state fMRI BOLD signal amplitude in the left angular gyrus in fibromyalgia patients
Source: Front Psychol. 2015 Jul 22;6:1051. doi: 10.3389/fpsyg.2015.01051 (PMC4510313; doi:10.3389/fpsyg.2015.01051)
Supplement: Supplementary file 3 [file Table1.DOCX]

**Supplementary Table 1. Comorbidities and medication.**

| *ID* | *Comorbidity* | *Medications* |
| --- | --- | --- |
| 1 | Glaucoma | NSAID |
|  | HPV infection |  |
| 2 | Migraine | Pregabalin |
|  | Allergic rhinitis | Procaine |
|  |  | SSRI |
|  | Vertigo |  |
| 3 | Neurocardiogenic syncope |  |
|  | Cleft palate (treated) |  |
| 4 | NR | TCA |
|  |  | NSAID |
| 5 | NR | TCA |
|  |  | NSAID |
| 6 | NR | Benzodiazepine |
|  |  | Opioid analgesic |
|  |  | TCA |
|  |  | NSAID |
| 7 | Hypothyroidism | Fludcortisone |
|  |  | SSRI |
|  |  | Levothyroxine |
|  |  | Melatonin |
|  |  | Benzodiazepine |
|  |  | Glucosamine-Chondroitin |
| 8 | NR | Gabapentin |
|  |  | Valproate |
|  |  | NSAID |
|  |  | Ranitidine |
|  |  | SSRI |
| 9 | NR | NR |
| 10 | NR | Benzodiazepine |
| 11 | Hypothyroidism | Levothyroxine |
|  |  | Benzodiazepine |
| 12 | NR | Gabapentin |
|  |  | NSAID |
| 13 | NR | Chondritin |
|  |  | Eletriptan HBr |
|  |  | Nabilone |
| 15 | NR | Opioid analgesic |
|  |  | Aspirin (preventive) |
|  |  | NSAID |
|  |  | DMARD |
| 17 | HTN | TCA |
|  |  | NSAID |
| 18 | HTN | TCA |
|  | DM2 | Losartan |
|  | Hypothyroidism | Eletriptan HBr |
|  | IBS | NSAID |
|  | Migraine | Levothyroxine |
| 19 | HTN | Enalapril maleate |
|  | Hypothyroidism | Levothyroxine |
|  |  | TCA |
|  |  | Cyclobenzaprine |
|  |  | NSAID |
| 20 | NR | TCA |
|  |  | Benzodiazepine |
| 21 | NR | Hormone Replacement |
|  |  | Glucosamine-Chondroitin |
| 23 | NR | NR |

NR = Not reported, NSAID = Non-steroidal anti-inflammatory drug, SSRI = Selective serotonin reuptake inhibitor, TCA = Tricyclic antidepressant, DMARD = Disease-modifying antirheumatic drugs, HTN = Hypertension.
